# In Memoriam

**Published:** 1886-04

**Authors:** Hunter P. Cooper

**Affiliations:** Atlanta, Ga.


### IN MEMORIAM.

### DR. J. B. FICKLEN.

On the 3d of March the soul of this beloved physician left its home of clay and soared to its immortal rest. For many years he had suffered with diabetes mellitus, and though he tried every known method of treatment, he at last fell a victim to its ravages. Dr. Ficklen was born on April 30th, 1830, and was consequently in his fifty-sixth year at the time of his death. His father, Dr. Fielding Ficklen, left his native State, Virginia, in 1825, and having located in Washington, Ga., practiced medicine with great success until 1855. Dr. J. Burrell Ficklen, his oldest son, graduated in medicine at the University of Pennsylvania in 1853, and returned to Washington to practice. When the war came on, he promptly offered his services to the Confederacy, and was appointed surgeon to the 4th Georgia hospital in Richmond., Va., in which capacity he served with great distinction until the close of the war. After the surrender he turned his face homeward and settled down to his life-work. His great ability as a physician soon procured for him a large and lucrative practice, and in the course of a few years he was recognized as the leading doctor of Wilkes and the surrounding counties.

In 1869 he was married to Miss Julia Weems, the daughter of Dr. Walter H. Weems. The influence which his gentle Christian wife exerted upon him by the daily example of her blameless life bore its fruit, when he professed religion about three years ago and united himself with God's people.

The writer enjoyed the privilege of knowing him intimately, having been a student of medicine under his preceptorship, and hence had unusual facilities for judging of his character, both as a man and a physician. As a man he was noted for his vigorous mind, blunt, direct honesty, sincerity in friendship and detestation of hypocrisy. His opinions were never given hap-hazard, but with the deliberateness and decisiveness of a man who has carefully weighed the subject, and has a reason for his conclusion.

He won his success as a physician by the sheer force of his intellect. He was a man of few words, never indulged in small talk, and had a certain brusqueness of manner which would have been fatal to the success of a man of less talent. His powers of reasoning and observation made him not only an exact diagnostician, but a successful therapeutist. As a surgeon his courage and skill were backed by a profound knowledge of anatomy. When once his mind was made up to a plan of treatment, there was no wavering or hesitation in carrying it out, and this courage gave his patients the most absolute confidence in him. A mind so active as his finds its pleasure in constant employment. As a consequence he was a student all his life, and having a retentive memory, he stored up a vast amount of medical lore. He added to his library all important works as soon as they were published, and in this way kept thoroughly abreast of the times. It is much to be deplored that he has left so little of his experience on record; had he used his pen more, not only would his reputation have been more extended, but the profession would have derived great benefit from his writings. His untimely end is mourned by hundreds who hold him in loving remembrance as physician and friend.

Hunter P. Cooper, M. D.

*Atlanta, Ga.*

